# Design of a Low-Power Embedded System Based on a SoC-FPGA and the Honeybee Search Algorithm for Real-Time Video Tracking

**DOI:** 10.3390/s22031280

**Published:** 2022-02-08

**Authors:** Carlos Soubervielle-Montalvo, Oscar E. Perez-Cham, Cesar Puente, Emilio J. Gonzalez-Galvan, Gustavo Olague, Carlos A. Aguirre-Salado, Juan C. Cuevas-Tello, Luis J. Ontanon-Garcia

**Affiliations:** 1 Facultad de Ingeniería, Universidad Autónoma de San Luis Potosí (UASLP), Dr. Manuel Nava No. 8, Zona Universitaria Poniente, San Luis Potosí 78290, San Luis Potosí, Mexico; oscar.cham@uaslp.mx (O.E.P.-C.); cesar.puente@uaslp.mx (C.P.); egonzale@uaslp.mx (E.J.G.-G.); carlos.aguirre@uaslp.mx (C.A.A.-S.); cuevas@uaslp.mx (J.C.C.-T.); 2 Centro de Investigación Científica y de Educación Superior de Ensenada (CICESE), Carretera Ensenada-Tijuana No. 3918, Zona Playitas, Ensenada 22860, Baja California, Mexico; olague@cicese.mx; 3 Coordinación Académica Región Altiplano Oeste, Universidad Autónoma de San Luis Potosí (UASLP), Carretera Salinas-Santo Domingo No. 200, Salinas de Hidalgo 78600, San Luis Potosí, Mexico; luis.ontanon@uaslp.mx

**Keywords:** heterogeneous computing, meta-heuristic, video tracking, system-on-chip, field-programmable gate array, evolutionary computing, swarm intelligence, embedded system design, graphics processing unit, computer vision

## Abstract

Video tracking involves detecting previously designated objects of interest within a sequence of image frames. It can be applied in robotics, unmanned vehicles, and automation, among other fields of interest. Video tracking is still regarded as an open problem due to a number of obstacles that still need to be overcome, including the need for high precision and real-time results, as well as portability and low-power demands. This work presents the design, implementation and assessment of a low-power embedded system based on an SoC-FPGA platform and the honeybee search algorithm (HSA) for real-time video tracking. HSA is a meta-heuristic that combines evolutionary computing and swarm intelligence techniques. Our findings demonstrated that the combination of SoC-FPGA and HSA reduced the consumption of computational resources, allowing real-time multiprocessing without a reduction in precision, and with the advantage of lower power consumption, which enabled portability. A starker difference was observed when measuring the power consumption. The proposed SoC-FPGA system consumed about 5 Watts, whereas the CPU-GPU system required more than 200 Watts. A general recommendation obtained from this research is to use SoC-FPGA over CPU-GPU to work with meta-heuristics in computer vision applications when an embedded solution is required.

## 1. Introduction

One of the goals of computer vision algorithms is the statistical analysis of the raw units of data contained in digital images or videos to allow the automated interpretation of visual information [1]. As in many other tasks, computer vision requires a massive number of operations on a massive amount of data. Video tracking is one of the computer vision problems that has become more relevant as a result of its application in various industries and technologies, such as robotics, unmanned vehicles, and automation, among others [2]. The video tracking problem essentially requires one to quantify the similarity of two ordered sets of data, with each component of these sets known as a pixel. Early video tracking approaches relied heavily on linear algebra and statistical examination to recognize patterns. Many different algorithms, colloquially known as video trackers, have been proposed in an attempt to solve the video tracking problem. Efforts to develop and improve video trackers are still ongoing; however, the problem remains open as perfect accuracy has not yet been achieved [3]. Additionally, each image in a digital video contains a massive amount of pixel data, resulting in high computational costs whenever they are inspected [4].

The purpose of video tracking, sometimes called object tracking, is to locate an object within a given image frame. It should be noted that the location of the object requires the detection of the same object in a previous frame [5]. Therefore, using a similarity measure, such as mean shift, normalized cross-correlation (NCC), regional mutual information (RMI), or zero mean normalized cross-correlation (ZNCC), is an elemental component to perform video tracking. Many of these techniques are still at the heart of newer proposals that use similar image matching criteria but which incorporate machine learning techniques, such as the support-vector machine of the Struck video tracker [6] or the decision tree of the tracker proposed by Asgarizadeh et al. [7] to choose between NCC and RMI as a similarity measure. The majority of today’s most accurate video trackers, such as the SiamMask video tracker [8], are powered by convolutional neural networks [3]. However, these proposals have drawbacks that stem from the training phase, which introduces issues such as sampling bias, vulnerability to adversarial attacks, and a high demand for memory and computational resources to store and process considerable amounts of training data [9,10]. Another approach follows a symbolic learning approach, using an artificial dorsal stream as the backbone while incorporating genetic programming to build a robust machine learning paradigm [11]. The first results show the trustworthiness of results in such a computational visual process [12,13,14].

Although the video tracking community focuses on accuracy issues, other factors such as video tracker speed must also be considered [15]. For example, some studies in the literature have used tools such as heterogeneous computing architectures and population-based meta-heuristics to improve efficiency rather than efficacy [16,17]. A heterogeneous computing system is any computing system that coordinates two or more different types of processing elements. The processing elements that can be used are typically central processing units (CPUs), graphics processing units (GPUs), digital signal processors (DSP), or field-programmable gate arrays (FPGAs), but they can be applied to any other microprocessor design [18]. These systems are developed to meet the increasing demand for higher computational performance as well as lower energy consumption, total size or area of the hardware elements, and monetary prices [19,20]. The same motivations have driven manufacturers to innovate with the system-on-chip (SoC) paradigm. An SoC is any complex integrated circuit that encapsulates all of the elements of a fully functional system on the same chip [21]. A standard SoC includes a CPU, memory, hardware acceleration units, and peripheral interfaces.

Using different processing elements benefits different aspects of a problem. A CPU is better suited for control-intensive operations such as searching, parsing, and sorting, whereas a GPU is better suited for image processing or other data-intensive tasks [22]. The FPGA is a versatile tool, capable of performing both parallel and sequential tasks while consuming less power, allowing the system design to be customized according to the requirements of the problem to solve. On the other hand, it introduces new challenges, such as the requirement for hardware/software co-design methodologies [23]. Currently, CPU-GPU and CPU-FPGA platforms, particularly SoC-FPGA, are the most researched heterogeneous systems [20]. CPU-GPU-FPGA platforms are gaining attention as they enable developers to map computations to specialized processing units that can perform their tasks more efficiently. However, there are still issues with the CPU-GPU-FPGA platform, such as the increased task scheduling complexity, which is currently being investigated [24].

This work studies the benefits of the honeybee search algorithm (HSA) [25,26] in combination with the use of an SoC-FPGA platform to design and implement a low-power embedded system for real-time video tracking. HSA is a meta-heuristic that replaces the mechanical and meticulous inspection of all possible answers with a mindful search procedure that reduces resource waste. It is based on evolutionary algorithms that use biological evolution as a source of inspiration to perform intelligent directed searches over a given feasible space. These methods simulate how the fittest individuals survive and reproduce for several generations until a stopping condition is met [27]. HSA also belongs to the field of swarm intelligence meta-heuristics, which are inspired by the collective behavior of biological agents throughout their lifespan [28]. Swarm intelligence meta-heuristics are inspired by social animals such as ants, bees, fireflies, birds, and fish, including particle swarm optimization (PSO) [29] and artificial bee colony [30] and ant colony optimization [31]. Many of these meta-heuristics have been proven to help with real-world applications and benchmarking functions. However, no standardized decision criteria exist to select one over the others for any given problem.

### 1.1. Related Work

After the literature review, we found that there have been several research projects that explore the integration of population-based meta-heuristics and heterogeneous computing platforms applied to real-world problems. A common issue with swarm intelligence meta-heuristics implementations on CPU-GPU heterogeneous systems is that when global synchronization is required for certain activities, it cannot be achieved because GPU hardware is not designed to do so [32]. Depending on the model, GPU core synchronization is only possible between groups that belong to the same logic block. The synchronization issue significantly reduces the computational resources that can be used effectively to parallelize a swarm intelligence meta-heuristic using this type of heterogeneous system [33,34]. Commonly, the CPU and GPU are separate hardware products in a CPU-GPU heterogeneous system, whereas an SoC-FPGA is a single chip that contains the processing system (PS) and the programmable logic (PL). Because these processing elements are so close together, they can communicate more quickly while using less energy, with the advantage of using the custom advanced extensible interface (AXI) bus [35]. In other words, the internal communication of an SoC-FPGA device should outperform CPU-GPU communication via USB or PCI Express. Furthermore, the PS is typically built with ARM technology, which is a reliable and energy-efficient standard RISC architecture, widely used in embedded solutions centered on signal and image processing, such as video tracking [36].

Table 1 shows some works in the state of the art that belong to this research niche. A subtle insight is that most authors focus on the particle swarm optimization (PSO) meta-heuristic or variants of this approach. Other works use an FPGA device to study feasibility of their proposals, but this research attempts to contrast SoC-FPGA and CPU-GPU heterogeneous systems to find which is the best option in this particular setting. Additionally, the comparison with other state-of-the-art video trackers in terms of their speed and power consumption is also performed in order to highlight the advantages of our proposal. Another distinction with respect to our research is that evaluation is commonly performed using toy data, whereas this work uses the Amsterdam Library of Ordinary Videos (ALOV) benchmark, which provides 314 video sequences with annotated initialization and ground truth data [37]. These video sequences are arranged in 14 categories that pose different challenges for video trackers. The number and diversity of video sequences makes this benchmark a good alternative for testing the performance of video tracker proposals. The work of Morsi et al. [16] is particularly interesting because the application is video tracking, but the work uses only FPGA. Another interesting work is that of Nogueira and Barboza [17] because it studies SoC-FPGA and CPU-GPU heterogeneous computing platforms; however, the application is not specific as it focuses on continuous optimization problems.

In short, there are several opportunity areas. The literature has not sufficiently reported the advantages and disadvantages of CPU-GPU and SoC-FPGA platforms when used to implement meta-heuristics in computer vision problems. The main contribution of this work is the novel design of a low-power HSA-based embedded system developed on an SoC-FPGA platform for real-time video tracking, with the aim of determining whether a specific video tracker can be accelerated without a loss of accuracy. Moreover, the performance in terms of accuracy is evaluated using the ALOV benchmark in order to confirm that there are no negative effects compared to the employed base similarity measure (ZNCC). This work contributes with (1) the proposed workflow for the design of an embedded system based on an automaton that describes the behavior of a honeybee searching for food [38] and an SoC-FPGA platform; (2) the design, implementation, and evaluation of a low-power embedded system that performs real-time video tracking using the combination of the HSA meta-heuristic and an SoC-FPGA platform, which was designed from scratch using the co-design methodology [39]; (3) the comparison of our proposal with a CPU-GPU HSA-based video tracking system [34] in terms of speed, energy consumption, accuracy, as well as portability; and finally, (4) the contrast with other state-of-the-art video trackers in terms of speed and energy consumption to highlight the advantages of our proposal in these terms.

**Table 1:** Brief summary of the related work which focuses on other publications that deal with heterogeneous computing systems and meta-heuristics.

Source	Year	Meta-Heuristic	Heterogeneous Platform	Application
Palermo et al. [40]	2008	Discrete PSO	SoC-FPGA	Multi-objective Design, space exploration
Tsai et al. [41]	2010	DNA algorithm	SoC-FPGA	Fire extinguishing
Morsi et al. [16]	2013	PSO	FPGA	Structural similarity index for video tracking
Rodriguez and Moreno [42]	2015	GA (genetic algorithm)	FPGA	Motion estimation with particle filter
Elkhani et al. [43]	2018	Multi-objective binary PSO	CPU-GPU	Feature selection and classification
Perez-Cham et al. [34]	2020	HSA	CPU-GPU	ZNCC for video tracking
Nogueira and Barboza [17]	2020	GRASP (greedy randomized adaptive search procedure)	SoC-FPGA, CPU-GPU	Continuous optimization problems

### 1.2. Organization

The main sections of this paper are organized as follows. Section 2 goes into detail about the materials and methods used to enable other researchers to recreate and build on the results that are reported. Then, Section 3 delves into the specific experiments that were performed and the results that were obtained. The interpretation of those results, along with general observations, are discussed in Section 4. Finally, a summary of the highlights and future work is presented in Section 5.

## 2. Materials and Methods

This section details the hardware and software tools used to design and implement the proposed real-time video tracking embedded system. The vast majority of these materials are associated with the ZC706 SoC-FPGA evaluation board, which is used in this work. This evaluation board contains a Zynq-7000 SoC-FPGA [44], which has been successfully used for image processing [45], signal processing [46], Internet of Things [47], and other applications. The Zynq-7000 processing system (PS) is made up of a dual-core ARM Cortex-A9 processor, with a Kintex-7 FPGA [48] serving as the SoC programmable logic (PL). The main advantage of using an SoC-FPGA over an FPGA is the ease of use of a CPU to perform specific tasks, such as dealing with external memory devices and reading and preprocessing image files. The section will also provide details on the evaluation of the fitness function employed by the HSA meta-heuristic which is the ZNCC similarity measure; this fitness or objective function guides the exploration of the solution space to find a suboptimal approximation to the peak function value which maximizes the similarity between the juxtaposed image patches. The last section is dedicated to detailing the proposed workflow based on hardware/software co-design methodologies and focuses on how to take advantage of the HSA meta-heuristic and the SoC-FPGA platform.

### 2.1. Programmable Fabric

Given their notable emulation capabilities and availability, FPGA devices are currently the most popular type of programmable logic devices (PLDs). Using hardware description languages (HDLs) such as VHDL, these devices can be reconfigured to behave like a described hardware design [49]. The main top-level components of an FPGA are independently configurable and are included as follows: configurable logic blocks (CLBs), input/output blocks (IOBs), and switching matrices. CLB components implement internal logic behavior, IOB components receive and transmit external data, and all of these components are interconnected as specified by switching matrices. The CLB is composed of several smaller components that are used to implement custom logic. This section describes the primary components used by the ZC706 evaluation board’s PL as the foundation for larger custom logic designs. The proposed custom system design makes use of a diverse set of Zynq-7000 PL components, the vast majority of which are categorized either as LUT (LookUp Table) or DSP48.

The LUT is the core component of the CLB, and each LUT is actually an array of one-bit multiplexers [50] with several one-bit inputs capable of implementing any arbitrary Boolean function with that number of inputs. In addition, each LUT contains a flip-flop that can be used to introduce sequential behavior if needed. The Boolean function of a given LUT is expressed as a truth table, which is stored in SRAM (static RAM) registers as needed. An FPGA has a large number of LUT components that allow for the implementation of complex combinational logic circuits using switching matrices. The DSP48 block is a physical ALU embedded in the fabric of Xilinx FPGA devices that is made up of three different blocks. This element consists of an add/subtract unit linked to a multiplier, which is linked to a final add/subtract/accumulate engine. The multiplier block can accept a single input value of up to 25 bits and a second input value of up to 18 bits. Given that multipliers consume a significant amount of programmable logic fabric resources (LUT components), using a DSP can result in area and power savings, while allowing the designer to use the programmable fabric for other tasks. The ZC706 board’s Zynq-7000 SoC has a LUT count of 218,600 and 900 DPS48 blocks that can be used as the designer sees fit.

### 2.2. Soft Intellectual Property Cores

An intellectual property (IP) core is a reusable unit of logic, cell, or integrated circuit layout design that is the intellectual property of one party (such as Xilinx). Soft IP cores are frequently available as HDL files. Designers can easily implement those same cores on any FPGA to accelerate system design. This section goes over some of the IP cores used in the design of the proposed system described in Section 3.1. Xilinx Vivado 2017.3 was the IDE used in the system’s development process, and it provided access to the used soft IP cores. Some vital Soft IP cores are those related to AXI communication between PS and PL.

Soft IP cores that perform relatively complex arithmetic operations, such as division or square root, are also important components. The system presented in this paper employs the CORDIC Soft IP core to compute square roots as needed to compute a given ZNCC value (Section 3.1). The CORDIC algorithm, also known as Volder’s algorithm [51], is a method that can be implemented using low-level software or hardware that uses simple shift-add operations to perform a series of two-by-two matrix multiplications to compute trigonometric functions, hyperbolic functions, or square roots. However, some significant limitations of this resource include the inability to use the DPS48 blocks available in the SoC and the maximum input size of 48 bits. Xilinx’s Divider soft IP core supports three different division algorithms: LUTMult, Radix-2, and High Radix [52]. The Radix-2 algorithm was chosen from among the three available algorithms because it returns the actual integer quotient of the division. LUTMult is a fast solution that takes advantage of the DSP48 blocks, but it consumes memory resources that should be used to store image data.

### 2.3. The Honeybee Search Algorithm Meta-Heuristic

The honeybee search algorithm (HSA) is similar to other honeybee-inspired meta-heuristics that mimic honeybees’ search for food. The metaphor of flying individuals searching for food is useful for the general image processing problem because the search space is large in both cases and hints of the optimal solution are widely dispersed [38]. In that sense, HSA is heavily influenced by the dynamic flies heuristic of Boumaza and Louchet [53], but with a greater emphasis on coordinated work. HSA differs from other approaches in that it is a hybrid proposal that combines techniques from swarm intelligence and evolutionary algorithms for real-valued multidimensional optimization problems [25].

Swarm intelligence and evolutionary algorithms developed independently, but they share similar terminology and can be generalized as population-based meta-heuristics [54]. These meta-heuristics are inspired by life and biological processes in their approaches to solving general optimization problems. In the terminology of population-based meta-heuristics, a specific solution is referred to as an individual. These methods are typically iterative, with an initial population of individuals that changes over time, guided by the fitness function. A generation is the term used to describe each iteration of this process. Several individuals compete metaphorically against one another, and it is determined which ones are better than others based on their fitness. The best individuals are rewarded, whereas the worst individuals are punished. This process continues for several generations until the population reaches a point where it is close to the optimal value. The (μ+λ) evolution strategy (ES) [55] is the fundamental evolutionary algorithm in HSA. This evolutionary algorithm has two populations: a parent population of μ individuals and an offspring population of λ individuals. The (μ+λ)-ES is an elitist ES, which means that individuals from the parent population can be chosen to be part of the population of the next generation.

**Figure 1:**
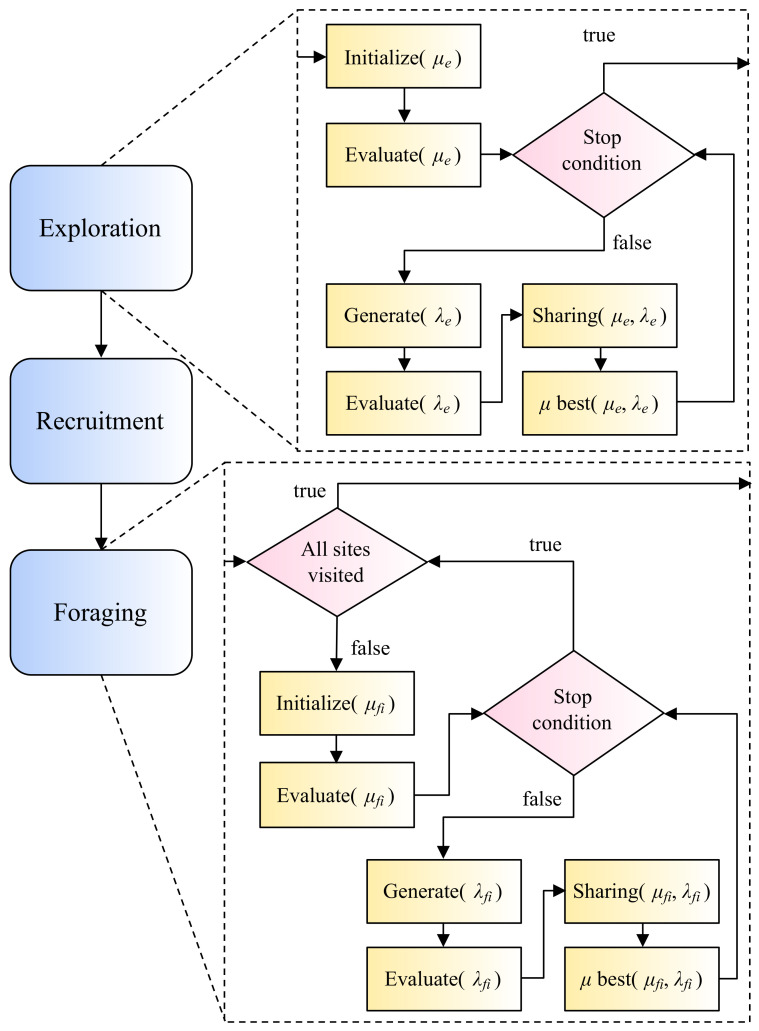
HSA has three main phases: exploration, recruitment, and foraging. The exploration and foraging phases are based on the canonical (μ+λ)-ES but implement different evolutionary operators.

Exploration, recruitment, and foraging (Figure 1) are the three main stages of the original HSA proposal [26]. The exploration phase of the optimization procedure begins with an initial inspection of the search space using the described modified version of the (μ+λ)-ES. The recruitment stage emulates how honeybees coordinate their efforts to find food sources. Real honeybees communicate with one another through their dance language, which allows them to spread very reliable and complex information [56]. These data are later used by their peers to make group decisions. Foraging is the final phase, which involves a series of (μ+λ)-ES subphases but with smaller search spaces and a larger number of individuals. These new limits are based on the previous phase’s resource planning and distribution. It was initially proposed that HSA should be run on a single CPU. However, Perez-Cham et al. [34] recently demonstrated that the same meta-heuristic could be executed on a CPU-GPU platform, with some benefits and drawbacks reported. This work applies the findings of that work to propose a workflow for designing another HSA implementation that uses an SoC-PFGA platform in an attempt to overcome the limitations observed with the restrictive CPU-GPU platform. Another recent publication [38] proposes an alternative representation of HSA using automata and formal languages theory. That representation is the basis for the proposed workflow (Section 2.5), facilitating the proper design process of the real-time video tracking embedded system (Section 3.1).

#### 2.3.1. The Evolution Strategy of HSA

The differences between the HSA (μ+λ)-ES and the canonical (μ+λ)-ES are mainly related to the generation of the λ offspring population. HSA replaces the ordinary mutation operator of (μ+λ)-ES, which is applied by adding normally distributed random values to the individual. Instead, HSA uses the polynomial mutation operator [55]. Another variation is that the common (μ+λ)-ES does not use any crossover operator, whereas HSA uses the simulated binary crossover (SBX) operator [57]. Futhermore, to avoid local optimam HSA randomly generates some of the members of the λ population. These three subdivisions of the λ population are called α or mutant sons, β or crossover sons, and γ or random sons. To respect the common ES terminology α, β, and γ are also the scalar variables that determine the size of the populations in such a manner that λ=α+β+γ. Depending on the specific problem, HSA may use the sharing operator of Goldberg and Richardson [58] that causes the effect of dispersion on the population instead of clustering. This is useful whenever a diverse population is required. It is important to mention that the populations of the exploration phase are labeled $\mu_{e}$, $\lambda_{e}$, $\alpha_{e}$, $\beta_{e}$, and $\gamma_{e}$. The foraging populations have their own names, $\mu_{f}$, $\lambda_{f}$, $\alpha_{f}$, $\beta_{f}$, and $\gamma_{f}$, to be discernible.

#### 2.3.2. Polynomial Mutation

This kind of mutation operator applies variation using polynomial distribution perturbation [59]. The mutation operation modifies a parent solution $x_{a}$ to generate a child solution $x_{b}$. Individuals are *k*-dimensional vectors, and the *i*-th dimension has a lower boundary $L_{i}$ and an upper boundary $U_{i}$. Equations (1) and (2) are used to get the *i*-th component of individual $x_{b}$, where $P_{\delta }$ is obtained using a uniformly distributed random variable ρ that ranges from 0 to 1, and a user-defined non-negative value $\eta_{m}$ that characterizes the probability distribution function.

(1) $$\begin{array}{c} x_{bi}=x_{ai}+\left(U_{i}-L_{i}\right)\cdot P_{\delta } \end{array}$$

(2) $$\begin{array}{c} P_{\delta }=\left\{\begin{array}{cc} {\left(2\rho \right)}^{1/(\eta_{m}+1)}-1 & \mathrm{if}\rho <0.5\\ 1-{\left[2\left(1-\rho \right)\right]}^{1/(\eta_{m}+1)} & \mathrm{otherwise} \end{array}\right. \end{array}$$

#### 2.3.3. Simulated Binary Crossover

The SBX operator [57] emulates the working principle of the single-point crossover operator on binary strings used by genetic algorithms. This operator receives two parent solutions $x_{a}$ and $x_{b}$ and uses them to create two children solutions $x_{c}$ and $x_{d}$. Equations (3)–(5) are used to generate the *i*-th component of *k*-dimensional vectors $x_{c}$ and $x_{d}$, where $P_{\beta }$ depends on a uniformly distributed random variable ρ that ranges from 0 to 1, and on a user-defined non-negative value $\eta_{c}$ that characterizes the probability distribution function.

(3) $$\begin{array}{c} x_{ci}=0.5\left[\left(1+P_{\beta }\right)x_{ai}+\left(1-P_{\beta }\right)x_{bi}\right] \end{array}$$

(4) $$\begin{array}{c} x_{di}=0.5\left[\left(1-P_{\beta }\right)x_{ai}+\left(1+P_{\beta }\right)x_{bi}\right] \end{array}$$

(5) $$\begin{array}{c} P_{\beta }=\left\{\begin{array}{cc} {\left(2\rho \right)}^{1/(\eta_{c}+1)} & \mathrm{if}\;\rho <0.5\\ {\left(\frac{1}{2(1-\rho )}\right)}^{1/(\eta_{c}+1)} & \mathrm{otherwise} \end{array}\right. \end{array}$$

#### 2.3.4. Recruitment Distribution

Equation (6) is used during the recruitment phase to distribute the individuals of the $\mu_{f}$ population into smaller groups $\mu_{fi}$ of size $r_{i}$ each. There is one group of $r_{i}$ individuals for every individual $x_{i}$ of the $\mu_{e}$ population. The size $r_{i}$ depends on the fitness value $f(x_{i})$ of the individual $x_{i}$ and the total sum of the fitness values of all the $\mu_{e}$ individuals. The individual $x_{i}$ that has the greatest $f(x_{i})$ will also have the greatest value of $r_{i}$. Metaphorically, the fittest exploration individual gets to recruit the largest number of peers from the foraging population to help in the exploitation of the resource that was found during the exploration phase.

(6) $$\begin{array}{c} r_{i}=\left\lfloor \frac{f(x_{i})}{\sum_{j=1}^{\mu_{e}}f\left(x_{j}\right)}\times \mu_{f}\right\rfloor \end{array}$$

#### 2.3.5. Zero-Mean Normalized Cross-Correlation as Fitness Function

Zero-mean normalized cross-correlation (ZNCC) is a method used to determine how similar two ordered groupings of data are. This similarity grade is expressed as a scalar real number ranging from −1 to 1 [60]. If the ZNCC function returns 0, it signifies that there is no correlation or resemblance between the two sets of data. There is a degree of correlation or resemblance between the compared groups of data if the absolute ZNCC value is larger than zero. Negative ZNCC values, on the other hand, show that the relationship between the two sets of data is inversely proportional.

ZNCC is a widely used method in computer vision problems, and there have been recent efforts to simplify or speed up its calculation [61]. If a region within an image file is translated as a two-dimensional array of values, ZNCC can be utilized for video tracking or other computer vision applications. These values of the two-dimensional arrays are commonly grayscale pixels stored as integers ranging from 0 to 255 (8-bits). In this work, ZNCC is used as the HSA fitness function because ZNCC remains a reliable, deterministic, non-trained legacy tracker proposal that has been successfully interpreted as an objective function as it has a well-defined domain that can be translated into a search space [38].

Equations (7) through (12) detail the calculation of ZNCC, where (p,q) is a spatial coordinate within a given image frame *I*; *t* is an image template; $I_{w}\times I_{h}$ is the size of *I*; $\bar{t}$ is the mean of *t*; (u,v) is a spatial coordinate inside *t* with top left corner (0,0) and bottom right corner $\left(t_{w}-1,t_{h}-1\right)$; $\bar{I}\left(p,q\right)$ is the mean of a region of *I* that has the same size as *t*; ZCC is the zero mean cross correlation of an image patch; and SSE is the sum of squared errors of an image patch. ZNCC values of different coordinates are compared to find the spatial coordinate (p,q), where a given image template *t* is most certainly located within an image frame *I*. The original search approach is exhaustive, meaning the full search space is thoroughly inspected. However, the well-defined equations can be repurposed as a fitness function for two-dimensional individuals.

(7) $$\begin{array}{c} \bar{t}=\frac{1}{t_{w}\times t_{h}}\cdot \sum_{u=0}^{t_{w}-1}\sum_{v=0}^{t_{h}-1}t\left(u,v\right) \end{array}$$

(8) $$\begin{array}{c} \bar{I}\left(p,q\right)=\frac{1}{t_{w}\times t_{h}}\cdot \sum_{u=0}^{t_{w}-1}\sum_{v=0}^{t_{h}-1}I\left(u+p,v+q\right) \end{array}$$

(9) $$\begin{array}{c} \mathrm{ZCC}\left(p,q\right)=\sum_{u=0}^{t_{w}-1}\sum_{v=0}^{t_{h}-1}\left[I\left(u+p,v+q\right)-\bar{I}\left(p,q\right)\right]\left[t\left(u,v\right)-\bar{t}\right] \end{array}$$

(10) $$\begin{array}{c} {\mathrm{SSE}}_{I}\left(p,q\right)=\sum_{u=0}^{t_{w}-1}\sum_{v=0}^{t_{h}-1}{\left[I\left(u+p,v+q\right)-\bar{I}\left(p,q\right)\right]}^{2} \end{array}$$

(11) $$\begin{array}{c} {\mathrm{SSE}}_{t}=\sum_{u=0}^{t_{w}-1}\sum_{v=0}^{t_{h}-1}{\left[t\left(u,v\right)-\bar{t}\right]}^{2} \end{array}$$

(12) $$\begin{array}{c} \mathrm{ZNCC}\left(p,q\right)=\frac{\mathrm{ZCC}(p,q)}{\sqrt{{\mathrm{SSE}}_{I}\left(p,q\right)\cdot {\mathrm{SSE}}_{t}}} \end{array}$$

### 2.4. Evaluation with the Amsterdam Library of Ordinary Videos

The Amsterdam Library of Ordinary Videos (ALOV) was presented by Smeulders et al. [37] in order to allow the evaluation and comparison of video trackers. Three hundred and fourteen video sequences with annotated initialization and ground-truth data are considered part of this benchmark, many of which are also present on other benchmarks. These video sequences are classified into 14 different categories, each with its own set of problems for video trackers. Because of the large number and variety of video sequences, this benchmark is a suitable choice to test the robustness of video trackers. Another appealing feature of the ALOV benchmark is its concentration on common video sequences generated by ordinary camera equipment in real-world situations. Frequently, the video sequences of other benchmarks contain alternative pixel data, including thermal, infrared, or depth information, which present greater levels of challenge [3].

A video tracker’s output is typically a rectangular area that encompasses the suspected position of the object of interest; this area is referred to as the video tracker’s truth. However, the video tracker’s truth does not have to be the actual location of the object of interest; that area is referred to as the ground truth. The amount of accuracy of the video tracker response can be geometrically translated as IoU=intersectionarea÷unionarea if the intersection and union areas of truth and ground truth can be measured. This notion of Intersection over Union (IoU) is utilized by many video tracking benchmarks, including the ALOV benchmark. A higher IoU value indicates greater accuracy, with a possible range from 0 to 1.

The ALOV benchmark recommends the detection of true positives and false positives in the evaluation process. This detection is based on a simple criterion, when IoU≥0.5, the truth of a video tracker is considered a true positive. Given that this criterion evaluates only one frame of a given video sequence, another metric, the F-score, is used to measure the outcome of the entire video. The F-score *F* is calculated using Equation (13), where $F_{a}$ is the number of true positives, $F_{b}$ is the count of false positives, and $F_{c}$ is the total of false negatives. Another ALOV benchmark recommendation is to use survival curve plots [62] to allow a simple visual inspection of the general trends observed in the F-score measurements of many video trackers with multiple video sequences. This same accuracy evaluation method is used in this paper.

(13) $$F=\frac{F_{a}}{F_{a}+\frac{1}{2}\left(F_{b}+F_{c}\right)}$$

### 2.5. Proposed Workflow

When a designer proposes cooperating hardware and software components in a single design effort, a hardware/software co-design methodology can be used [23]. This methodology arises from the radically different tasks of designing and optimizing hardware and software. Hardware developers are taught to think in terms of parallel space decomposition, whereas software developers are taught to think in terms of sequential time decomposition. However, the terms hardware and software can mean different things in different contexts. As a result, some authors redefine the co-design methodology as application partitioning and design using fixed and flexible components [39]. The used co-design methodology combines traditional top-down and bottom-up methodologies because both are used at some point during the design process. A top-down approach starts with a high-level view of the application and then investigates how to improve its performance by utilizing specific resources [63]. On the other hand, in a bottom-up approach, the developer begins with the resources and investigates how to integrate them into a working system as efficiently as possible [64].

**Figure 2:**
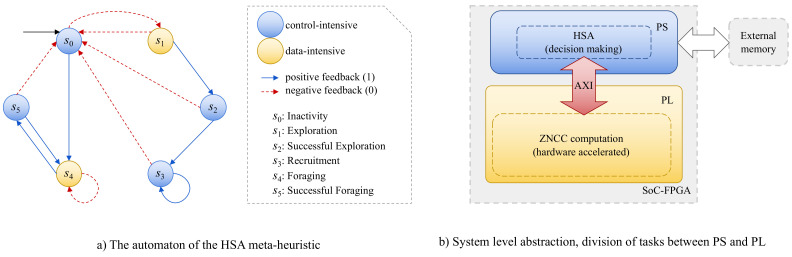
Proposed workflow to design the real-time video tracking system. (**a**) The automaton of Perez-Cham et al. [38] that formally describes the HSA meta-heuristic in an abstract non-architecture-specific manner. (**b**) System-level abstraction based on the HSA automaton that shows the division of tasks between PS and PL. The PS is in charge of control-intensive operations that are conducted by the HSA meta-heuristic (states $s_{0}$, $s_{2}$, $s_{3}$ and $s_{5}$), whereas the PL focuses on the acceleration of ZNCC via concurrency and parallel processing (states $s_{1}$ and $s_{4}$).

As previously stated in this paper, SoC-FPGA platforms are composed of two high-level components that are physically located on the same chip: the PL, which allows the emulation of a described electronic design; and the PS, which contains a dual-core ARM Cortex-A9 processor. Depending on the expected gain when parallelizing, we decided to perform certain HSA tasks in the PS and others in the PL in this case. The automaton (depicted in Figure 2a) and computational complexity analysis of Perez-Cham et al. [38] were critical tools that allowed the identification of the HSA meta-heuristic states or phases that benefit the most from hardware acceleration. Our conclusion was that the fitness function evaluation (ZNCC) is the part that incurs the most costs and has the greatest potential to be intuitively parallelized. As a result, this work proposes that the PS should perform HSA tasks related to intelligent coordinated decision making, whereas the PL should focus on ZNCC evaluation, corresponding to the exploration and foraging states (Figure 2b).

**Figure 3:**
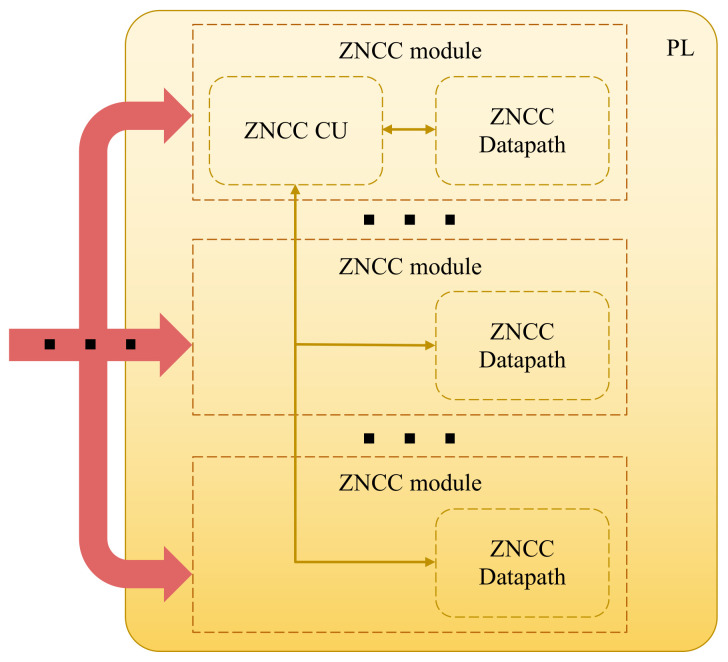
An illustration of how several ZNCC modules perform parallel processing, taking advantage of the capabilities of the PL. Each ZNCC module requires a datapath to perform operations simultaneously on different sets of input data, coordinated by a common control unit that oversees the execution of tasks following the SIMD parallel model.

Following the establishment of a division of labor between PS and PL, the next priority is to design a hardware module capable of computing ZNCC based on input data, which consists of two small image patches. Given the ZNCC computation’s sequential nature, this module should include a custom control unit and a custom datapath that performs the necessary arithmetic and logic operations while utilizing hardware concurrency whenever possible. Parallelization should also be possible through a coordinated array of ZNCC computation modules, each of which is dedicated to performing the same operations but on different input data (SIMD; single instruction, multiple data). The iterative design process for this ZNCC module employs both top-down and bottom-up approaches, adapting both the algebraic interpretation to the system’s basic low-level components and vice versa. An abstract representation of the array of ZNCC modules is illustrated in Figure 3.

Finally, communication between PS and PL should be treated as a distinct issue. To solve this problem, a thorough understanding of Xilinx’s AXI Bus solution is required. On SoC-FPGA platforms, communication between CPU and FPGA can be implemented in a variety of ways, but direct memory access (DMA) is best suited for data-intensive applications. When using DMA, the data transfer is handled by specialized hardware, whereas normal memory access is handled by the CPU [20]. This has two benefits: it speeds up the data transfer and frees up the CPU for other tasks. AXI CDMA is the most suitable solution and is thus used to communicate between the PS and PL components in the proposed system design. A detailed description of the implementation and operation of AXI CDMA is presented in Section 2.6.

The workflow described in this section served to guide the design process of the proposed embedded system, which is detailed in depth in the following section. As the niche is still being researched, the authors hope that other researchers will find this workflow proposal useful in proposing similar systems based on meta-heuristics and SoC-FPGA platforms.

### 2.6. Processing System and AXI Communication

The dual-core processor of the Zynq-7000 SoC can be programmed directly using assembly or low-level languages but this project uses a lightweight Linux operating system installation to allow the PS to serve as a development environment, along with compilation tools and useful software libraries such as OpenCV to easily manipulate image files. Furthermore, the operating system builds a logic layer that allows the use of board interfaces such as UART, USB, Ethernet, etc. A program running on Linux can interact with the physical memory that is directly used by the computer hardware, which is different to the virtual memory that is commonly used by programs. The interaction of a program with physical device memory is achieved through a file that is an image of the main memory, which can be located following the path /dev/mem, using the common root folder structure. In this case, the /dev/mem file is used to read and write data that are also manipulated by the PL, creating a bridge between PS and PL. AXI defines a protocol where several master and slave components interact using parallel, high-performance, synchronous, high-frequency transfer operations. Xilinx has adopted AXI4, AXI4-Lite, and AXI4-Stream [35] as the main communication interfaces that are used by their products. The PS can act as an AXI master or an AXI slave depending on the specific AXI peripherals that are implemented using the logic fabric.

As a reminder, the PS will focus on the activities of HSA that determine the intelligent behavior of the system, whereas the PL will focus on the acceleration of the fitness function (ZNCC). Communication between PS and PL is carried out through AXI interfaces of two different types: AXI GPIO and AXI CDMA. The simplest Xilinx AXI peripheral is the AXI GPIO (general purpose input/output) that directly reads or writes a register of 32 bits that are directly connected to physical hardware such as an output LED array, an input switch array, or other circuits implemented on the PL. AXI GPIO is used to send a 32-bit configuration word that is used to control the system design that is implemented using the PL. This configuration word is the length of the array of values that is sent as input. If the length value is zero, a global reset signal is fired.

ALOV image files are stored in JPEG format; however, they are read and converted to a one-dimensional array of grayscale unsigned integer values of 8 bits using software. These values are then transferred from the PS to the PL and stored using standard BRAM resources. The word size of the used BRAM is 32 bits, meaning that four grayscale values (of 1 byte each) can be written or read at once. The maximum capacity of BRAM resources is 8192 bytes ($2^{13}$) or 8 KiB and the data transfer is made using AXI CDMA blocks that read the image values from the PS and write them to the corresponding BRAM resource in the PL. The AXI CDMA (central DMA) peripheral can act as the PS AXI master, reading data from PS memory and writing it to other AXI slave peripherals’ memories. The request to perform a data transfer contains the following data: clear/work/standby configuration word (32 bits), starting source address in the memory device (32 bits), starting destiny address in the BRAM implemented on the PL (32 bits), and the count of bytes to be transferred (32 bits). When the AXI CDMA block receives those simple orders, the PS acts as the master device. However, once the AXI CDMA block starts working, it acts as the master device with the permission to read PS device memory directly. Since AXI CDMA is only dedicated to transferring data, this hardware is noticeably faster than the PS itself.

## 3. Results

This section considers the final proposed embedded system design as a result, since it was generated through an iterative design process using the general guidelines that were established in the proposed workflow. The experimental results that validate the proposed system are also presented in this section. Some opportunity areas for improvement have been identified and are discussed below in the Discussion and Conclusions sections.

### 3.1. Proposed Embedded System Design

**Figure 4:**
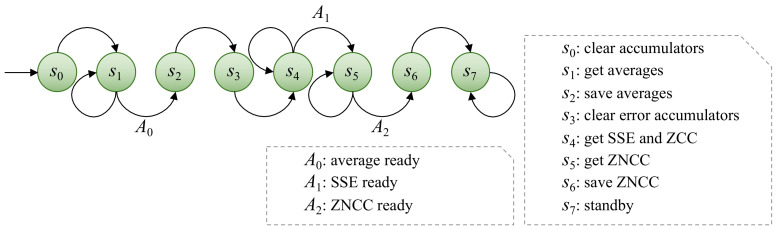
A visualization of the Moore machine of the ZNCC CU. This CU supervises the custom ZNCC module’s proper functioning. It has control over another CU that manages accumulation and datapath elemental blocks.

**Figure 5:**
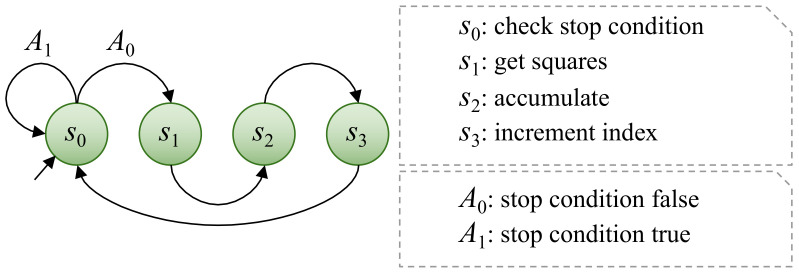
A Moore machine illustration describing the behavior of the accumulation CU. Summations constitute the vast majority of the sequential operations required to compute ZNCC, which is why the flow of those operations should be managed by means of a separate control unit.

This section explains how the proposed embedded system is designed using the workflow detailed above, and also how it works in a modular way. The following sections detail the hardware design implemented using the PL component. As a reminder, the PL focuses on the evaluation of ZNCC, given that it was previously identified as the bottle-neck, because it is the most computationally expensive task. A custom digital system design generally requires two top-level elements: the control unit and the datapath [65]. In order to facilitate the description of the proposed system, the description starts with the datapath, which is composed of elemental blocks that were created by combining the fixed components described in the previous sections. The average, SSE, and RSSE blocks are the primary blocks used to perform arithmetic and logic operations. These modules perform the necessary operations, but the sequential flow is controlled by finite state machines [66] implemented as control units. Multiple instances of these components can be implemented, allowing several ZNCC values to be computed at the same time. The main control unit, ZNCC CU (Figure 4) and the accumulation control Unit, CU (Figure 5) are defined below to explain the interrelations and data dependencies between the datapath elemental blocks.

### 3.2. Datapath

**Figure 6:**
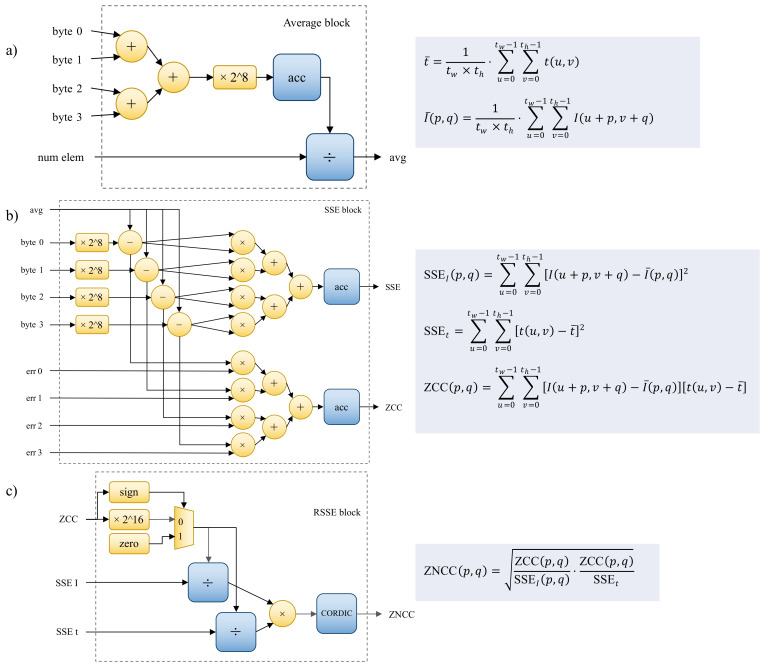
This figure shows how the elemental blocks of the datapath are built from available soft IP cores and basic low-level and logic arithmetic blocks, each of which is implemented using the programmable fabric of the FPGA. (**a**) The average block; (**b**) the SSE block; (**c**) the RSSE block.

This section explains the main building blocks of each ZNCC module’s datapath. The first block, the average block (Figure 6a), was designed using the bottom-up approach to calculate the average of a set of 8-bit positive integers. To adapt to the BRAM component that is used to store the array of input data, four input bytes are received at once. The resulting unsigned 8-bit integers are added using three simple binary adders. Then, using a hardwired shift left of 8 bits, the sum is multiplied by $2^{8}$. This sum is the input to the main sequential accumulator, which follows the instructions of the accumulation CU (Figure 5) and ZNCC CU (Figure 4). The accumulator’s 32-bit output is the dividend input of a sequential divider that employs the Radix-2 algorithm. The length of the array of numbers is the divisor input of the same divider block. The accumulation stop condition and the ZNCC CU provide the signal to begin working on the division. When the division is finished, an output signal is generated, which is the average done signal. The average is a 16-bit unsigned integer, stored in a register. This block’s components are all implemented with the LUT programmable fabric.

The SSE block (Figure 6b) was also designed using the bottom-up approach. It takes four input bytes and applies the same treatment as the average block to produce four 16-bit unsigned integers. To obtain the individual error values, the previously obtained corresponding average is substracted from each of the integer values. It is worth noting that some of these blocks output error values (differences from the average), which can be used by other blocks to calculate ZCC. The block that computes both SSE and ZCC has eight 17-bit multipliers, four of which are used to calculate squared errors and the remaining four are used to cross multiply the errors. The accumulation CU (Figure 5) and the ZNCC CU (Figure 4) control the 48-bit accumulators in both variations. The DSP48 components are used to implement the multipliers, whereas the rest of the blocks are LUT-based.

The RSSE block (Figure 6c) design process required a top-down approach. When working with integers, some complications arise, such as the loss of significant figures when the result of an operation has a decimal part. Floating-point operations, on the other hand, consume a lot of resources and are avoided in this design. This is why this system modifies ZNCC to always work with integers. The CORDIC block is used to compute the square root of a truncated integer given a 48-bit input integer. This work proposes using some simple algebraic manipulations to compute ZNCC more quickly without losing detail in the results; this is an example of how the function adapts to the available resources. Equation (14) demonstrates how to obtain the squared ZNCC value by performing two parallel divisions and one multiplication. ZCC values can be either positive or negative, which ultimately indicates if the detected correlation is inverse or not. This work proposes ignoring negative ZCC values, which are considered undesirable in the fitness function because they do not indicate a strong correlation of the compared image patches. This adjustment in the ZNCC calculation avoids confusion in this application and saves resources. A simple multiplexer is used to implement this, which outputs zero whenever a negative ZCC value is detected (Figure 6c).

(14) $$\begin{array}{c} {\mathrm{ZNCC}}^{2}\left(p,q\right)=\frac{\mathrm{ZCC}(p,q)\cdot \mathrm{ZCC}(p,q)}{{\mathrm{SSE}}_{I}\left(p,q\right)\cdot {\mathrm{SSE}}_{t}}=\frac{\mathrm{ZCC}(p,q)}{{\mathrm{SSE}}_{I}\left(p,q\right)}\cdot \frac{\mathrm{ZCC}(p,q)}{{\mathrm{SSE}}_{t}} \end{array}$$

To avoid the loss of resolution when the result is truncated, the ZCC input value of this block is multiplied by $2^{16}$ before calculating both divisions, as indicated in Figure 6c. The quotients of the dividing blocks are the inputs to a 24-bit multiplier, which is used to calculate the square of the ZNCC value, as indicated in Equation (14). A single CORDIC block starts working on the square root of the squared ZNCC value when the dividers emit a positive done signal. As a result, the output of the RSSE block is a positive integer ranging from 0 to $2^{16}$, or 0 to 10,000 in hexadecimal numbers, where a value of $2^{16}$ means total similarity between both input images (*t* and *I*). At least one of these blocks is required for the system to function, but several can be used simultaneously to obtain a large number of ZNCC values.

#### 3.2.1. Control Units

The main control unit is called ZNCC CU (Figure 4); it is in charge of the overall operation of the custom ZNCC module. It has authority over another CU that controls the accumulation and the datapath elemental blocks. The Moore machine used to describe the CU has eight states $S=\{s_{0},s_{1},s_{2},s_{3},s_{4},s_{5},s_{6},s_{7}\}$. The tasks that are performed in each of these states are: clear the average accumulators to start from zero ($s_{0}$); work to get averages using the accumulation CU ($s_{1}$); save the averages to use them later ($s_{2}$); clear the SSE and ZCC accumulators to start from zero ($s_{3}$); work to obtain the sums of squared error (SSE) and zero-mean cross-correlation (ZCC) values using the accumulation CU ($s_{4}$); work to compute the ZNCC value ($s_{5}$); save the ZNCC value ($s_{6}$); stand by and wait for a hard reset ($s_{7}$). The input alphabet *A* has three input characters: a signal that indicates that the average is ready ($A_{0}$), a signal that indicates that the SSE is ready ($A_{1}$), and a signal that indicates that the ZNCC value is ready ($A_{2}$).

Because several of the sequential operations required to calculate a ZNCC value are summations, the accumulation CU (Figure 5) was created to control their sequential flow. The same control unit is used to guide two different summation phases that are taken into account in the overall flow provided by the ZNCC CU. This control unit is used to perform sequential integer accumulation in two cases: to obtain sums that will be used to calculate averages, and to obtain SSE and ZCC values. All averages can be evaluated in parallel, but SSE and ZCC require averages to be computed first. As a result, the design takes into account two accumulation phases. The Moore machine that describes this CU has four states $S=\{s_{0},s_{1},s_{2},s_{3}\}$, where $s_{0}$ is the state in which the stop condition is checked. Furthermore, during state $s_{0}$, the CU waits for the BRAM to deliver the data that are currently being requested. The data received from the BRAM are then multiplied to obtain the square in state $s_{1}$, if necessary. When the CU is in the $s_{2}$ state, the next step is to accumulate the value or the squared value. Finally, in state $s_{3}$, a signal is provided to indicate that the process’s index should be incremented. There are only two input characters in the input alphabet *A*: the signal that indicates that the process is not complete ($A_{0}$) and the signal that indicates the opposite ($A_{1}$). These input characters are used to indicate when the stop condition has been met, which is that the index is greater than the length of the array. In general terms, the behavior of this CU is very similar to a for-loop in common programming.

#### 3.2.2. System Overview

The average, SSE and RSSE blocks, which were discussed in previous sections, are the main blocks utilized to conduct arithmetic and logic operations. These modules carry out the necessary tasks, but the sequential flow is managed by finite state machines, which are implemented as control units. The PL can be used to implement several instances of these components, allowing multiple ZNCC values to be computed at the same time. The designed system can be characterized as an SIMD device because it performs the same actions on multiple sources of input data.

**Figure 7:**
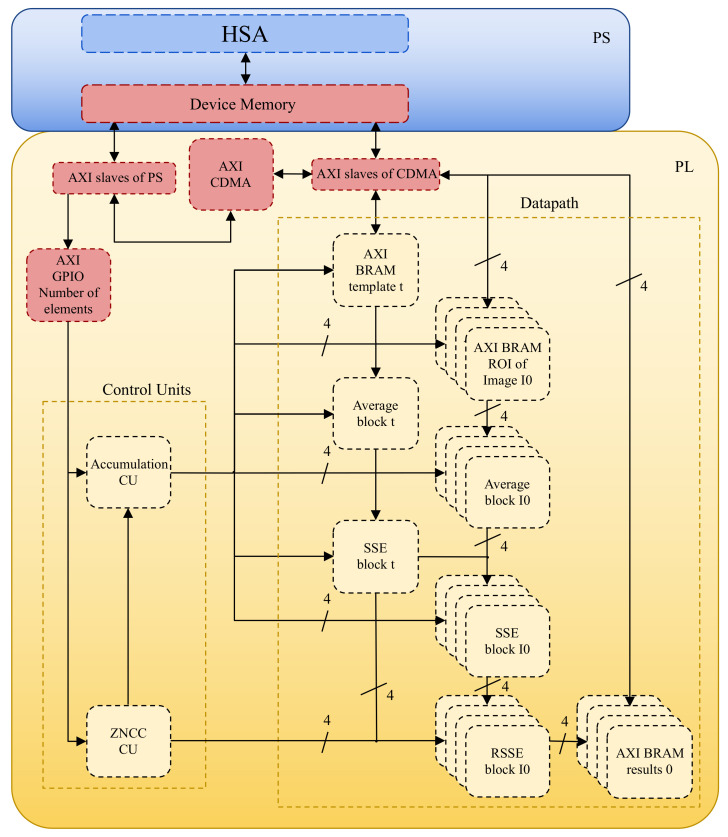
An illustration of the final interconnection of the ZNCC processing unit’s top-level blocks. Average blocks, SSE blocks, RSSE blocks, the accumulation CU, and the ZNCC CU are the key blocks that were implemented utilizing the PL. The communication between PS and PL is implemented using AXI, where CDMA is used to transfer image data and GPIO is used to transfer simple control data.

To compute four ZNCC values in parallel, the final iteration of the system (Figure 7) implements five average blocks, five SSE blocks, and four RSSE blocks. With the specific SoC-FPGA that is employed (Zynq-7000 XC7Z045), a higher degree of parallelization is theoretically conceivable as the full implemented solution makes use of 40% of the available LUT programmable fabric and 7% of the DSP blocks. However, this version of the proposed system fits the resources of all the SoC-FPGA devices that contain an FPGA that belongs to the Xilinx Kintex-7 product family [48], making this design reproducible with a wider variety of devices.

### 3.3. Experiments

A full description of the system that was used in the reported experiments is provided in Section 3.1; the top-level overview is illustrated in Figure 7. As a reminder, this system is built upon the ZC706 Evaluation Board that contains a Zynq-7000 SoC-FPGA, specifically a XC7Z045 device. Most experiments are based on the data and methods of the ALOV benchmark or certain selected video sequences; the description of how ALOV is used for evaluation can be found in Section 2.4.

#### 3.3.1. Calibration Test with a Static Image

By definition, real numbers ranging from −1 to 1 make up the range of values returned by the ZNCC function (Section 2.3.5). However, the custom hardware design proposed in this work modifies this range to make better use of the hardware resources. The following changes have been made: negative numbers are now ignored, the range has been adapted from 0 to $2^{16}$, and the values are now discrete integer numbers; in other words, the result values are binarized. This section describes a simple test intended to confirm that the modifications have no negative effects on the accuracy of the ZNCC tracker.

**Figure 8:**
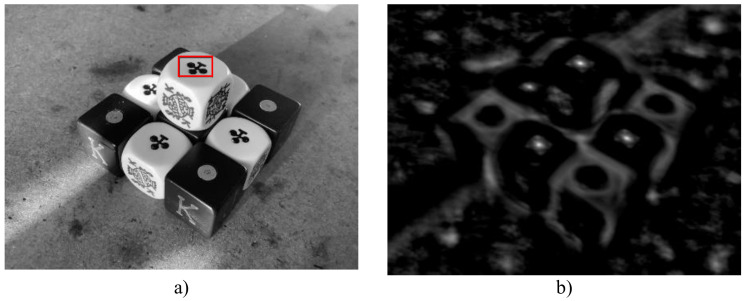
Visualization of the results of the calibration test. (**a**) A grayscale image with an instance of an ace of clover (object of interest) contained within a red bounding box. (**b**) A graphical representation of ZNCC values, with brighter spots representing the spatial location of the highest ZNCC values where similar objects are spotted.

The custom ZNCC unit was used in this test to perform the exhaustive search of a simple template within the same image frame where it was originally located. Figure 8a depicts the static image that was used. Given that the only point with 100 percent similarity is the same location as the initial object, the test is deemed successful. A closer look at the ZNCC values (Figure 8b) reveals that they are qualitatively correct because other similar instances of the object are also marked as bright spots.

#### 3.3.2. Testing the SoC-FPGA ZNCC-Based System with One Video Using Exhaustive Search

**Figure 9:**
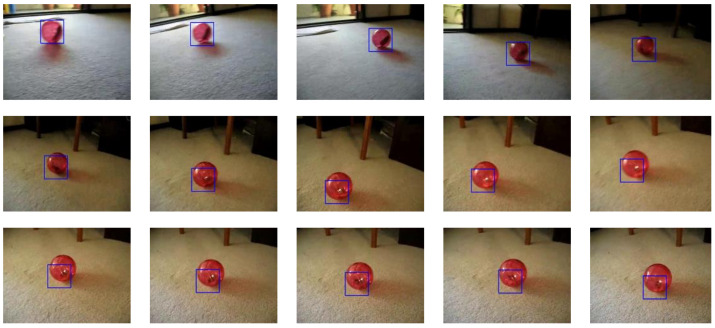
This figure displays some of the video frames that show how the tracker follows the object of interest (hamster ball). This test was made using the proposed embedded system to determine the feasibility of using it to accelerate an exhaustive search.

Considering that the previous test demonstrated that the proposed ZNCC unit can find an object with 100% similarity, this test aims to confirm that the video tracking results are as accurate as those obtained with the plain CPU-GPU heterogeneous system of Perez-Cham et al. [34], which is also exhaustive. Due to the high time-costs associated with an exhaustive search, this test employed only one video. The chosen video is the twenty-third video from the ALOV benchmark’s Light category, which follows a hamster ball as it rolls on the scene’s floor (Figure 9). The obtained F-score was 0.975, which is identical to the result obtained using the CPU-GPU heterogeneous system. This demonstrates that the proposed ZNCC unit can identify a visually similar object even if the similarity is not perfect in the ZNCC scale.

Nevertheless, it was observed that when using a GPU, the execution times of the exhaustive search were reduced. The CPU-GPU heterogeneous system took 0.0503 s per frame on average to process this video, whereas the SoC-FPGA with the proposed configuration had a time-cost of 5.0004 s per frame. The superiority of the CPU-GPU heterogeneous system is expected in this setting, given that the GPU is the ideal architecture for a naive exhaustive search and that the GPU’s full capacity is used in that case. The following examinations concentrate on the effects of the HSA meta-heuristic.

#### 3.3.3. CPU-GPU versus SoC-FPGA Using HSA

**Figure 10:**
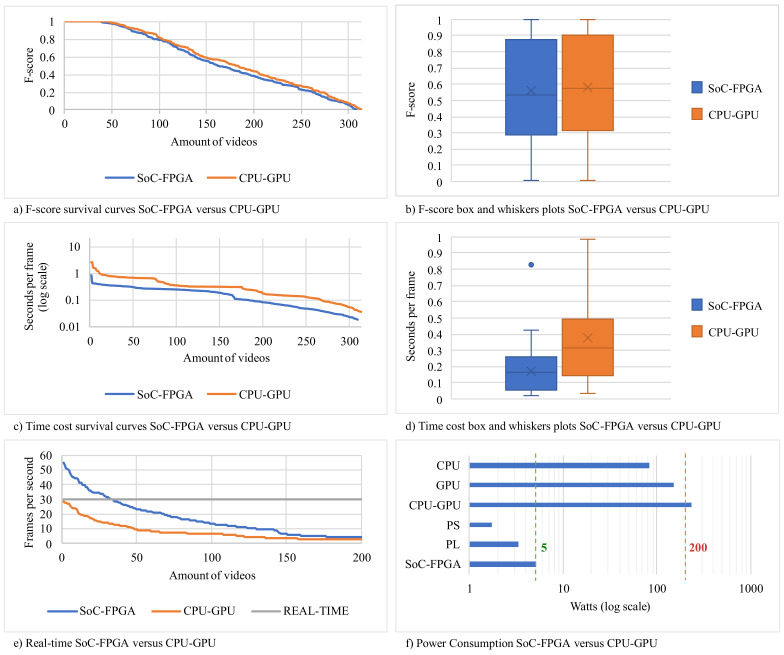
The compared video tracking systems use the same similarity measure (ZNCC) and the same meta-heuristic (HSA). The main difference between these proposals is the platform used to build the systems (SoC-FPGA or CPU-GPU). (**a**) SoC-FPGA versus CPU-GPU in terms of accuracy using survival curves. (**b**) SoC-FPGA versus CPU-GPU in terms of accuracy using box-and-whisker plots. (**c**) SoC-FPGA versus CPU-GPU in terms of time-costs using survival curves. (**d**) SoC-FPGA versus CPU-GPU in terms of time-costs using box-and-whisker plots. (**e**) SoC-FPGA versus CPU-GPU in terms of real-time video tracking. (**f**) SoC-FPGA versus CPU-GPU in terms of power consumption.

The SoC-FPGA heterogeneous system was used to process 309 ALOV benchmark videos (Section 2.4). As explained in Section 3.1, the PL computes ZNCC to be used as a fitness function, and the PS is in charge of the HSA meta-heuristic. The accuracy comparison using the F-score (Figure 10a,b) shows that there is only a minor difference between using the SoC-FPGA heterogeneous system and the CPU-GPU heterogeneous system. However, the difference is only 6.6 percent of the total standard deviation, which is insignificant. A Student’s *t*-test of two samples was used to compare the mean accuracy of CPU-GPU against SoC-FPGA. The resulting *p*-value of 41.19% (considerably greater than 5%) confirms the null hypothesis (means are the same). This indicates that the results of the implemented video tracking embedded system do not show any significant differences in accuracy.

The work of Perez-Cham et al. [34] provides a computational complexity analysis that provides evidence that the size of the object of interest in pixels has a significant effect on time-costs whenever HSA is used; this fact influences the following statements about the observed time-costs. In terms of time-costs, the SoC-FPGA outperformed the CPU-GPU (Figure 10c,d). The average time per frame (measured in seconds per frame) obtained with the SoC-FPGA was 0.1697, which is less than the average of 0.3729 obtained with the CPU-GPU heterogeneous system. Furthermore, the standard deviation of the time per frame measurements obtained using the SoC-FPGA was 0.1199, whereas the standard deviation of the CPU-GPU heterogeneous system was 0.3461, indicating that the SoC-FPGA was approximately 2.8 times more stable.

Using the SoC-FPGA increases the speed of this video tracker proposal and enables real-time processing with specific frame sizes (Figure 10e). Thirty-two videos (10.19%) from the ALOV dataset were processed in real-time; the videos were those where the size of the template was less than 3800 pixels. As a reminder, 30 frames per second was previously defined as the cutting edge of real-time video tracking, at least for this work. Furthermore, the SoC-FPGA consumed less power (Figure 10f). According to the hardware specifications, the CPU-GPU heterogeneous system consumed approximately 40 times more power than the SoC-FPGA heterogeneous system. This suggests that the use of an SoC-FPGA heterogeneous system combined with HSA is an efficient way to accelerate a video tracker.

#### 3.3.4. Comparison against State-of-the-Art Trackers in Terms of Time-Costs

The experimental results of Perez-Cham et al. [34] provide evidence that the measured accuracy of the CPU-GPU system is no match for recent state-of-the-art video tracker proposals such as Struck and SiamMask. This is related to the underlying similarity measure that is used (ZNCC). However, it was important for the development of this work to maintain a certain degree of complexity as the integration of SoC-FPGA and HSA was not a trivial matter. Additionally, the simplicity of using ZNCC as a similarity measure allowed a useful and thorough cross-platform study, which was already expounded in the previous section. This has been the primary goal of the ongoing research project that envisions a unified methodology to integrate meta-heuristics and SoC-FPGA platforms, which remains an unexplored field.

**Figure 11:**
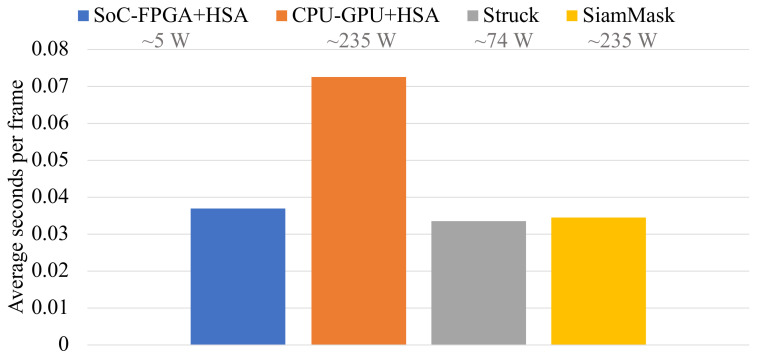
Comparison of the SoC-FPGA system against state-of-the-art trackers in terms of time-costs. The results of the CPU-GPU system are shown for contrast. Labels provide information about the estimated power consumption of the hardware that is typically used in each proposal. Note that the SoC-FPGA system is the only proposal that meets low-energy requirements.

With that in mind, it would be redundant to report that the low-power embedded system design for real-time video tracking presented in this work is not a suitable competitor in terms of accuracy, as it was demonstrated that the CPU-GPU system and the SoC-FPGA system deliver the same levels of accuracy. However, it should be noted that the new proposal is in fact close to becoming a strong contender in terms of time-costs, as shown in Figure 11. It should be noted that the time-costs of the original proposal based on CPU-GPU are substantially higher than those of the newer SoC-FPGA-based proposal and this trend should continue. Another important factor is power consumption and portability. Although Struck and SiamMask deliver outstanding accuracy, it is only possible to meet their energy and processing demands by using recent high-end mainstream CPU-GPU devices and computers.

## 4. Discussion

The scope of this project was to demonstrate that it is possible to develop an embedded solution for real-time video tracking with low power consumption and that it obtains reliable results, combining the benefits of the SoC-FPGA platforms and bioinspired meta-heuristics, which have been described in detail in previous sections of this work. The proposed system design uses 40% of the LUT (87,440) and 7% of the DSP48 (63) resources that are available on the ZC706 evaluation board, which allows scalability and portability, using similar platforms. In future works, we will consider redesigning the system to fit the full extension of resources of the ZC706, focusing on different aspects such as higher speed or even higher precision. Table 2 shows the properties of Zynq-7000 SoC-FPGA devices that include a Kintex-7 FPGA [48], to illustrate the posibility of portability and scalability employing different SoC-FPGA evaluation boards.

A common trend in embedded system design that employs FPGA technology is to select relatively simple computer vision algorithms, given the difficulty of translating them to hardware. A ZNCC tracker was selected to be used in this work because it has a low complexity, which allows a more feasible and functional design and implementation in the SoC-FPGA platform. As explained in Section 3.3.4, ZNCC was fundamental to this study due to its low complexity, which permitted cross-platform testing. Additionally, ZNCC reliably detects similarity and remains a feasible building block for mature video tracking solutions. Moreover, the ALOV benchmark [37] which was used initially considered ZNCC one of the contenders, given that it is a legacy tracker, and this allowed us to compare it against the original CPU approach. The accuracy levels that have been attained using recent video trackers such as Struck [6] and SiamMask [8] are superior to that of ZNCC. However, the proposed system obtains an average real-time result similar to those obtained for these state-of-the-art trackers, but with the advantages of lower power consumption and portability.

The precise definition of real-time image processing varies from 10 to 240 frames per second [15,67]. However, this work considers 30 frames per second as the minimum speed of a real-time tracking system as this measure is based on human visual perception. The speed of 30 fps can be achieved using the combination of HSA and SoC-FPGA, as shown in Figure 10b and Figure 11. However, it is only possible when the object of interest is contained on a rectangle with a size of 3800 pixels or lower. The only relationship we observed between videos that were processed in real-time and their category is that these videos were those in which the object was relatively small compared to the size of the frame, particularly in surveillance cameras. In embedded system design, it is very important to focus efforts on a well-defined specific application. According to the evaluation carried out with the ALOV benchmark, we concluded that the ideal use of the proposed system is in low-resolution videos used for surveillance, where it is intended to follow people or vehicles which have a small size with respect to the size of the frame, which is observed when the camera it is found at high points or far away.

The CPU-GPU architecture has some limitations, including the high time-costs of communication, the limited coordination of GPU components, and the fixed nature of the CPU-GPU architecture. The implemented solution based on CPU-GPU and HSA used only a fraction of the available GPU cores, and most of them remained idle. The CPU-GPU heterogeneous system was created to accelerate massive rendering operations. According to the experiments presented in this work, CPU-GPU is better suited to performing exhaustive searches. The SoC-FPGA allows a greater control of the datapath and control units to perform image multiprocessing. The modules of the atomic operations may be used as required by the specific problem. In the case of the fitness function (ZNCC), the problem was reassessed to fit the available resources in a bottom-up fashion. One of the few disadvantages of using SoC-FPGA is that a complex iterative co-design methodology is required, which demands a deep understanding of different computer architectures (dual core ARM, FPGA, IP cores, DPS, etc.) to find an optimal design that is effective, energy-efficient, and which requires less computational resources and processing time. However, introducing HSA simplifies the hardware design process. Given that HSA does not explore the full search space, it is not strictly necessary to transfer the full frame from PS to PL. In that sense, HSA reduces the demand on memory resources.

## 5. Conclusions

In summary, this work presented the novel design, implementation, and evaluation of a low-power embedded system based on an SoC-FPGA platform and the HSA meta-heuristic for real-time video tracking. The main conclusions of this work are listed below.

1. An original workflow was proposed for the design of a low-power embedded system for real-time video tracking, based on an automaton that describes the behavior of a honeybee searching for food [38] and an SoC-FPGA platform. The workflow described in Section 2.5 served to guide the design process of the proposed embedded system. As the niche is still being researched, we hope that other researchers will find this workflow proposal useful in order to suggest similar systems based on meta-heuristics and SoC-FPGA platforms. It is useful to identify which parts of the meta-heuristic are control-intensive and which ones are data-intensive to identify the labors of PS and PL.
2. A novel design, implementation, and evaluation of a low-power embedded system that performs real-time video tracking by combining HSA meta-heuristics and an SoC-FPGA platform was presented. Several benefits were observed using HSA in combination with SoC-FPGA for video tracking. The SoC-FPGA allows a greater control of the modules of the atomic operations. In the case of the fitness function (ZNCC), the problem was reassessed to fit the available resources in a bottom-up fashion. The time-costs are lower using an SoC-FPGA, which makes real-time processing possible. Furthermore, SoC-FPGA makes it possible to process a greater frame size in real time. Additionally, SoC-FPGA allows noticeably lower power consumption than CPU-GPU platforms and a greater portability. The experiments demonstrated that HSA can successfully be used to accelerate ZNCC for video tracking using SoC-FPGA without negative effects on accuracy.
3. The comparison of our SoC-FPGA HSA-based proposal with a CPU-GPU HSA-based video tracking system [34] in terms of speed, energy consumption, accuracy, as well as portability, allowed the identification of the limitations of the CPU-GPU platform in this context. These limitations were the high time-costs of communication, the limited coordination of GPU components, and the fixed nature of the CPU-GPU architecture. We recommend using CPU-GPU over SoC-FPGA only if the problem requires an exhaustive search and the solution does not require portability and consider using a meta-heuristic over a GPU whenever possible. The greatest reduction in time-costs was observed when HSA was used in combination with SoC-FPGA.The results of the evaluation provide evidence that the combination of SoC-FPGA platforms and meta-heuristics is promising as it enables the creation of portable, energy efficient, fast, and effective systems.
4. The results of the comparison with other state-of-the-art video trackers (Struck and SiamMask) showed that our proposal has the advantages of lower power consumption and portability, while maintaining similar processing speeds. On the other hand, Struck and SiamMask deliver outstanding accuracy, but they require high-end mainstream CPU-GPU devices and computers with high energy consumption. In this sense, the proposals of this work demonstrate that studying how to properly exploit the efficiency of the SoC-FPGA platforms in combination with meta-heuristics will bring substantial benefits to video tracking, other computer vision applications, and computational optimization in general.

Taking those findings into account, we propose the following future work to describe the direction of our research, considering the corresponding novel design, implementation, and evaluation tasks.

1. To improve the system that was designed to use the full capacity of the SoC-FPGA. The current proposal uses 40% of the LUT components, and 7% of the available DSP blocks. Additionally, we aim to exploit the possibility of reconfiguration, which allows the designer to propose many different designs that solve the same problem but with varying degrees of sequential and parallel behavior.
2. To verify whether HSA may be implemented using the PL of the SoC-FPGA. The current proposal uses the PL to compute the fitness function, but the general decision-making process is executed using the PS. Further experiments should be performed to find the advantages and disadvantages of using the PL to run HSA.
3. To use other fitness functions to replace or complement ZNCC. The canonical ZNCC tracker is currently not a viable contender against state-of-the-art trackers in terms of accuracy. However, it served as a starting point to study the advantages of using HSA and different heterogeneous systems for video tracking given its relative simplicity in comparison to newer proposals such as Struck [6] and SiamMask [8].
4. To use the combination of HSA and SoC-FPGA platforms in other CV applications. The results of using HSA for video tracking showed positive results. This motivates us to study the effect of HSA on other CV applications or on specific variations of the tested problems, for example, in face tracking and detection, or tracking based on infrared image data.

## Figures and Tables

**Table 2:** Properties of Zynq-7000 SoC-FPGA devices that include a Kintex-7 FPGA.

Device Name	Part Number	LUT Count	DSP Slices	Conforms to System Design
Z-7030	XC7Z030	78,600	400	Yes **
Z-7035	XC7Z035	171,900	900	Yes
Z-7045 *	XC7Z045	218,600	900	Yes
Z-7100	XC7Z100	277,400	2020	Yes

* This is the specific SoC-FPGA that was used in the experiments. ** The current design uses an LUT count of 87,440 and 63 DSP slices; an adaptation using more DSP slices instead of LUT components is feasible due to the excess of unused DSP slices.

## Data Availability

The ALOV dataset used in this work is available on http://alov300pp.joomlafree.it/.

## Abbreviations

The following abbreviations are used in this manuscript:

ALOV	Amsterdam Library of Ordinary Videos
ARM	Advanced RISC Machine
AXI	Advanced eXtensible Interface
CDMA	Central DMA
CLB	Configurable Logic Block
CU	Control Unit
CPU	Central Processing Unit
DMA	Direct Memory Access
DSP	Digital Signal Processor
ES	Evolution Strategy
FPGA	Field-Programmable Gate Array
GA	Genetic Algorithms
GPU	Graphics Processing Unit
GRASP	Greedy Randomized Adaptive Search Procedure
HDL	Hardware Description Languages
HSA	Honeybee Search Algorithm
IOB	Input/Output Blocks
LUT	LookUp Table
NCC	Normalized Cross-Correlation
PL	Programmable Logic
PLD	Programmable Logic Devices
PS	Processing System
PSO	Particle Swarm Optimization
RISC	Reduced Instruction Set Computer
RMI	Regional Mutual Information
RSSE	Root SSE
SIMD	Single Instruction, Multiple Data
SoC	System-on-Chip
SSE	Sum of Squared Errors
SRAM	Static RAM
ZNCC	Zero-Mean Normalized Cross-Correlation
